# Focal Segmental Glomerulosclerosis Followed by Granuloma and Preceding T-Cell Lymphoma by 46 Months: A Continuation Process or Coincidence

**DOI:** 10.7759/cureus.31847

**Published:** 2022-11-23

**Authors:** Samah Tahri, Houda Bachir, Siham Hamaz, Ismail Belefqih, Samia Malki, Amal Bennani, Habiba Alaoui, Khalid Serraj

**Affiliations:** 1 Internal Medicine Department, Immunohematology and Cellular Therapy Laboratory, University Hospital Mohammed VI, Faculty of Medicine and Pharmacy, Mohammed First University, Oujda, MAR; 2 Anatomy and Pathological Cytology Department, Faculty of Medicine and Pharmacy, Mohammed First University, Oujda, MAR

**Keywords:** corticosteroids, chemotherapy, paraneoplastic syndrome, peripheral t-cell lymphoma not otherwise specified, granulomatous inflammation, focal segmental glomerulosclerosis

## Abstract

Focal segmental glomerulosclerosis is a severe renal disease with a complex and unclear pathophysiology. Nephrotic syndrome is the clinical presentation of this renal disease. The recurrence of the disease after renal transplantation and the remission obtained after immune-adsorption treatment illustrate the implication of a circulating factor that requires characterization. Granulomatous inflammation is a tissue reaction caused by several conditions, including neoplastic diseases. In the literature, focal segmental glomerulosclerosis and granulomatous inflammation have both been associated with lymphoma. We report the case of a 56-year-old woman who initially developed focal and segmental glomerulosclerosis. After one year, the granulomatous inflammation was treated as tuberculosis infection and then as sarcoidosis. Finally, after another year, non-specified peripheral T-cell lymphoma was diagnosed.

## Introduction

Focal and segmental glomerulosclerosis (FSGS) is a common non-familial form of nephrotic syndrome in which the causative flaw remains undetermined. Several studies have suggested that FSGS is associated with the presence of circulating permeability factor and immune system disorder.

Granulomatous inflammation is a form of response to chronic inflammation caused by several assaulting agents, such as infection, autoimmunity disorder, toxic or allergic substances, and neoplastic diseases. The identification and classification of granulomatous inflammation patterns is helpful in narrowing differential diagnoses; nonetheless, a thorough clinicopathological review is required, especially in non-infectious etiologies.

Peripheral T-cell lymphoma not otherwise specified (PTCL-NOS) is a highly heterogeneous group of mature post-thymic T-cell lymphomas and the most common subtype. It requires a diagnosis of exclusion, a so-called wastebasket diagnosis, because it assembles entities that are not further classifiable into any of the other distinct entities.

Although an association between FSGS and lymphoma, especially Hodgkin’s lymphoma but rarely non-Hodgkin’s lymphoma, has been reported, there are no reported cases associated with PTCL-NOS.

The same has proven true for granulomatous inflammation, which has frequently been reported in association with neoplastic diseases, such as Hodgkin’s lymphoma and dendritic-cell or histiocytic lymphoma, but never with PTCL-NOS lymphoma.

As far as we know, this is the first case report to highlight the exceptional association between FSGS, granulomatous inflammation, and PTCL-NOS.

## Case presentation

We report the case of a 54-year-old woman with no medical or surgical history, and, specifically, a lack of diabetes, hypertension, or tuberculosis contagion.

In January 2018, the patient was consulted for generalized edema. The clinical examination found normal blood pressure (120/70 mmHg), normal heart and respiratory rate, afebrile temperature, no clinical signs of right heart or liver failure, but noted to have multiple lymphadenopathy at the bilateral cervical level. In addition, an otorhinolaryngological examination showed no abnormalities. A urine strip revealed the presence of protein but no blood. The blood count and smear were perfectly normal. A 24-hour urine test confirmed proteinuria at 4 g/24 h, hypoalbuminemia at 23 g/l, normal creatinine at 6 mg/l, and urea at 0.23 g/l. A cytobacteriological test of the urine was normal. Both a renal biopsy and cervical adenectomy were performed in response to the nephrotic syndrome and cervical lymphadenopathy. The renal biopsy presented an FSGS, and the adenectomy showed a non-specific chronic lymphadenitis. Before starting corticosteroids for the idiopathic nephrotic syndrome, a thorough assessment was performed for the chronic lymphadenitis. Viral hepatitis, immunodeficiency, and syphilitic serologies were negative, the search for tuberculosis infection was negative, and the cervical-thoraco-abdominopelvic computed tomography did not show any other suspected lesion but the cervical lymphadenopathy. The patient was treated with corticosteroids at 1 mg/kg/day of prednisone with a good response and total disappearance of the nephrotic syndrome and cervical adenopathy. Cessation of the corticosteroids was achieved within seven months.

**Figure 1:**
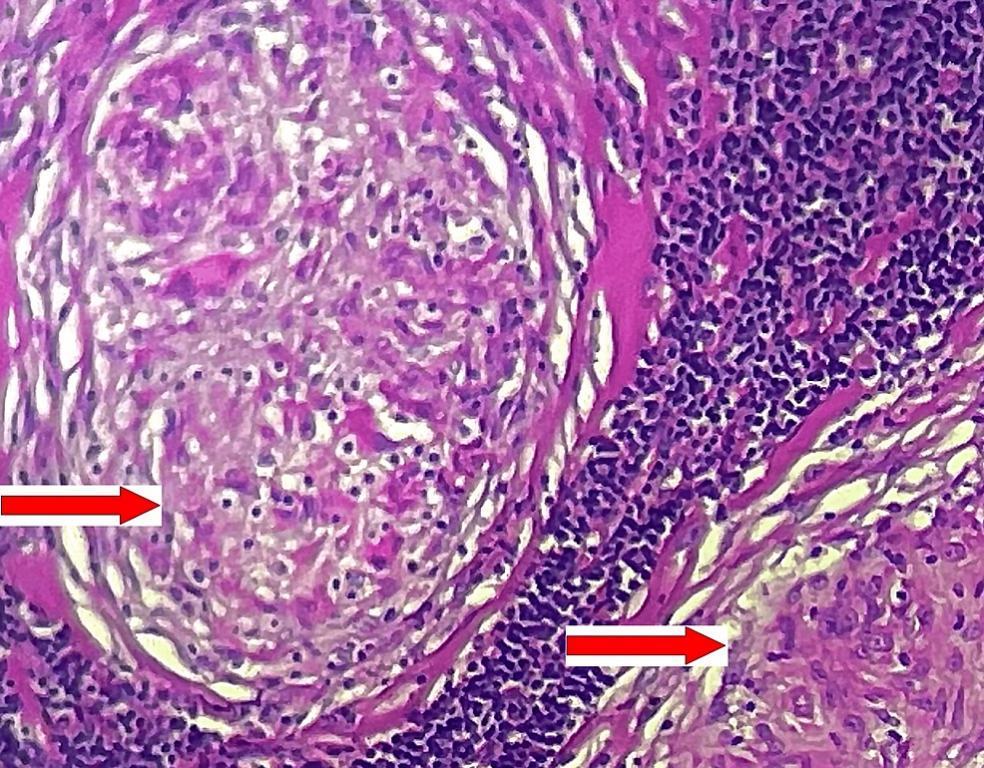
Microphotography showing ganglionic parenchyma whose architecture is erased and replaced by multiple epithelioid and gigantocellular granulomas without caseous necrosis (red arrows).

In July 2020, the patient was consulted for recurrence of lymphadenopathy with generalized invalidating pruritus. Upon examination, vital parameters were normal, and there was no edema nor anemic or infectious syndrome. Lymphadenopathy was present in the cervical and inguinal bilateral lymph nodes in association with splenomegaly. The blood count and smear were normal, serum albumin was correct at 45 g/l, 24-hour proteinuria was negative, renal and liver functions were correct, viral and syphilitic serologies were negative, C-reactive protein was negative, QuantiFERON-TB Gold assay was positive, and angiotensin-converting enzyme was normal at 35 U/l. In addition, serum protein electrophoresis showed hypogammaglobulinemia, but serum immunofixation was negative. A bone marrow biopsy was normal, and an adenectomy was performed, showing an epithelioid granulomatous adenitis with rare giant cells, a lack of caseous necrosis, and the absence of lymphomatous proliferation (Figure 1).

Based on these results, a decision was made to put the patient on antibacterial treatment (rifampicin, isoniazid, ethambutol, and pyrazinamide for two months, followed by rifampicin and isoniazid for five months). Evolution was marked by the persistence of lymphadenopathy and the worsening of the general state. Given the lack of a response to antibacterial treatment, the diagnosis of tuberculosis was unlikely, and the patient was put on corticosteroids with a good clinical response, size, and number reduction of lymphadenopathy and pruritus amelioration. During the digression of corticosteroids and prednisone at 5 mg/day, a relapse of the clinical presentation occurred.

**Figure 2:**
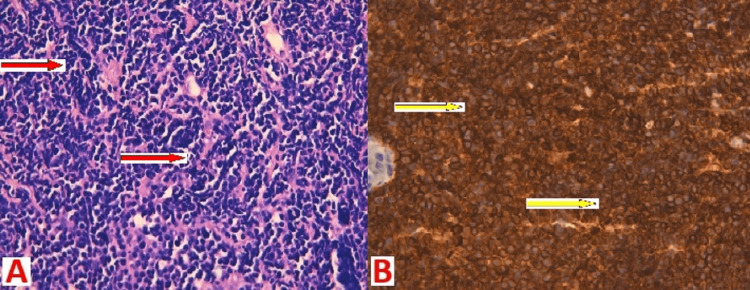
Microphotography showing tumor proliferation in layers, with small tumor cells lymphoid-like with rounded hyperchromatic nuclei and frequent cell mitosis (red arrows in A), positive membrane staining of tumor cells by anti-CD3 antibody (yellow arrows in B).

In October 2021, a third lymph node biopsy was performed. An anatomopathological and immunohistochemical study showed a PTCL-NOS. Tumor cells with anti-CD3, anti-CD4, and anti-CD8 antibodies were positively labeled. There were no signs of antibody labeling with anti-CD20, anti-CD5, anti-CD10, anti-CD30, anti-TDT, anti-BCL6, anti-PAX5, anti-ALK, or anti-EMA (Figure 2).

**Figure 3:**
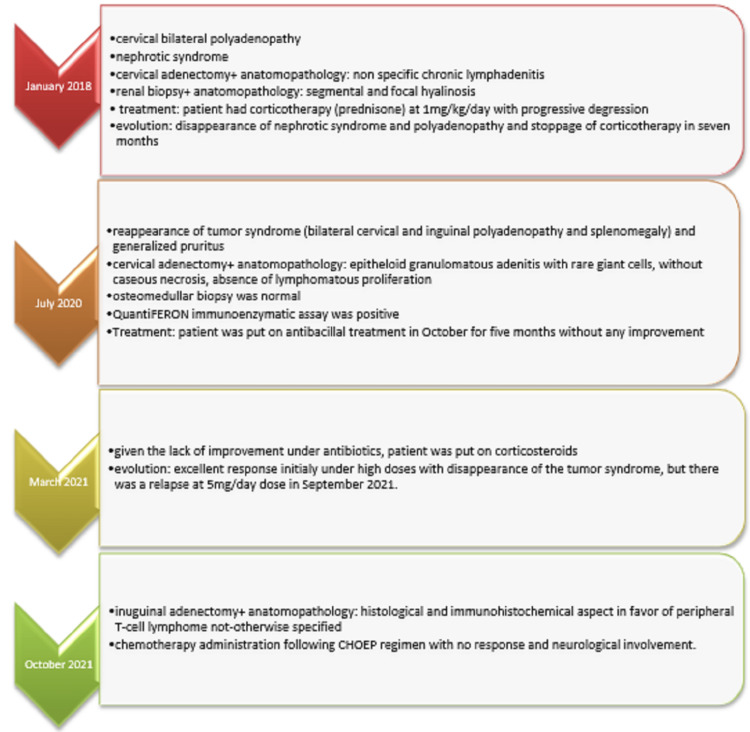
Timeline of clinical presentation, investigations and treatment

The disease was classified as stage IV due to medullary infiltration in the bone marrow biopsy. The patient was treated following a cyclophosphamide, hydroxyl daunorubicin, vincristine, etoposide, and prednisone (CHOEP) regimen for four 28-day cycles. Assessment after chemotherapy revealed disease progression. Clinically, the patient experienced severe headaches. Radiologically, the patient presented with an increased number of enlarged, deep lymphadenopathy. In front of the headaches, cerebral magnetic resonance imaging was performed, demonstrating a left parietal and right temporal pachymeningeal thickening associated with a hypersignal of the cerebellum and left Rolandic circumvolution. A lumbar puncture showed a clear fluid liquid, hypoglycorrhachia, hyperproteinorachia, the presence of 122 elements with 70% neutrophils, and the presence of tumor cells. The patient underwent high-dose methotrexate-based chemotherapy without intrathecal administration due to thrombopenia and died of a neurological disorder two days after the chemotherapy administration. The timeline of the clinical presentation, investigations, and treatment is presented in Figure 3.

## Discussion

FSGS represents the main cause of nephrotic syndrome in the United States (35%) [1]. It is less frequent in Europe, with an incidence rate of 12% in Spain in 2010 [2]. In Africa, no data have been reported concerning the epidemiology of this glomerulopathy. In Morocco, according to the registry of glomerulopathy and epidemiological transition, FSGS represents 9.1% of all glomerulopathy etiologies [3].

FSGS is characterized by its morphological heterogeneity, which probably reflects its varied pathogenic mechanisms. The Columbia classification of FSGS distinguishes five types of lesions according to their topographies in the glomerulus and the nature of the associated endocapillary and extracapillary damages as follows: nonspecific, cellular, perihilar, tubular pole, and flocculus collapse. On immunofluorescence, IgM and C3 deposits are observed in hyaline depositions. The main abnormality observable on electron microscopy is the deletion of the pedicels of podocytes [4]. Among the several etiologies, the most common is viral infection, with HIV virus at the top of the list, followed by parvovirus B19, cytomegalovirus, and Epstein-Barr virus [5]. FSGS can occur secondary to drug abuse such as heroin consumption and toxicity of drugs such as bisphosphonate; interferon alpha, gamma, and beta; anti-calcineurins; mTOR (mammalian target of rapamycin) inhibitors; and VEGF (vascular endothelial growth factor) inhibitors [5]. Adaptive FSGS is the consequence of either the reduction of the number of nephrons or mechanical constraints inducing stress on initially normal nephrons, such as obesity or thalassemia [6]. Several genetic studies have identified genetic mutations involved in 8% of FSGS etiologies in adults. These affected genes encode proteins of the slit diaphragm and the molecules regulating the actin cytoskeleton such as WT-1 (Wilms' tumor suppressor gene 1), NPHS1 (Nephrin), NPHS2 (podocin), ACTN4 (alpha-actinin-4), TRPC6 (transient receptor potential cation channel 6), CD2AP (CD2-associated protein), PLCE1 (phospholipase C epsilon 1), and INF2 (inverted formin 2) [7-14]. Idiopathic or primitive FSGS is a common form of non-familial nephrotic syndrome in which the causative defect is unknown. However, various arguments are in favor of a possible circulating factor and/or lymphocyte dysregulation, making FSGS a systemic disease. The implication of the immune system was suggested in face of the disease outbreaks following viral infections or vaccination. In addition, the fact that the disease outbreaks could be controlled by immunosuppressive therapy suggests a role for T cells and/or macrophages. Finally, the outbreak control by immunoabsorptions and B lymphocyte sensitivity to calcineurin inhibitors may suggest an involvement of B lymphocytes in the disease genesis, either directly or involving a T/B collaboration [5]. In the case of our patient, the investigation for nephrotic syndrome confirmed the diagnosis of idiopathic FSGS.

Nephrotic syndrome associated with hematological malignancies has been reported in absence of renal vein thrombosis or amyloidosis. This association was most frequently found in patients with Hodgkin's lymphoma but rarely found in patients with non-Hodgkin's lymphoma, especially those with T-cell lymphoma. The first case of FSGS associated to T-cell lymphoma was reported in 1981 by Belghiti et al. [15]. Two cases of cutaneous T-cell lymphoma associated with FSGS were reported in 1998 by Cather et al., which suggest that glomerular injury may be related to interleukin 2 expression [16].

Granulomatous inflammation is a histological pattern of tissue reaction to a chronic inflammation caused by various conditions, including infections and autoimmune, toxic, allergic, drug-induced, and neoplastic conditions. Multiple histological patterns can narrow the clinical differential diagnosis, including foreign-body, necrotizing, non-necrotizing, suppurative, and diffuse histiocytic reactions. Granulomas can precede the histological findings of lymphoma and mimic non-necrotizing granulomatous etiologies [17].

According to the World Health Organization (WHO), peripheral T-cell lymphoma, not otherwise specified (PTCL-NOS), is a diagnostic category within the highly heterogeneous group of mature post-thymic (hence “peripheral”) T-cell malignancies. The WHO classification divides PTCLs into nodal, extranodal, and leukemic types, each with several entities. Those which are no longer classifiable in a more distinct disease entity are called not otherwise specified [18]. This heterogenous category of PTCLs, the so-called wastebasket, represents the most frequent subtype, reflecting the fact that a large percentage of neoplasms in the PTCL spectrum still require accurate characterization [18]. The patients' median age was 60 years, with a sex ratio of 1.9 in favor of male predominance [19]. A study of the racial trends in the incidence of PTCL-NOS showed higher rates in blacks than in all other races [20]. PTCL-NOS are predominantly nodal lymphomas that can have extranodal sites. The most common site is the skin and gastrointestinal tract, and the coexistence of an extranodal disease with a nodal disease was found in 49% of cases [18]. Four prognosis models have been reported to be useful for patients with PCTL-NOS: the International Prognostic Index (IPI), the Prognostic Index for T-cell lymphoma (PIT), the modified PIT (m-PIT), and the International PTCL project (IPTCLP) score [21]. In a retrospective study that compared the four models, IPTCLP score was found to be the best measure of overall survival (OS) in a multivariate analysis [21]. Late-stage disease is the rule at presentation, with up to 60% of patients in stage IV and more than half of patients with high risk according to their prognostic scores [22]. Anthracycline-based chemotherapy regimens remain the most frequently used first-line therapy for patients with PTCL-NOS, with an overall response rate of 50%, a complete response rate of 20%, and a poor long-term survival, with a median three-year OS of nearly 30% [22]. In our case, the patient was diagnosed at stage IV at presentation. She was treated with combination chemotherapy following the CHOEP regimen (cyclophosphamide, hydroxyl daunorubicin, vincristine, etoposide, and prednisone). She survived only six months after being diagnosed with PCTL-NOS.

## Conclusions

FSGS remains a complex disease, and further study is needed to identify its exact pathogenetic pattern, thus opening up new therapeutic prospects. Histologically identified granulomatous inflammation is an effective predictor of diagnostic etiology. In the literature, both FSGS and granulomatous inflammation have been associated independently to hematological malignancies, but more often to Hodgkin's disease and rarely to PCTL-NOS. The present case is the first reported case of the coexistence of the three entities, which suggests the link between these abnormalities and the need to investigate further to eliminate the neoplastic etiology of FSGS or granulomatous inflammation.
